# Targeting steroid receptor coactivators for the treatment of benign female reproductive disorders

**DOI:** 10.1530/EC-25-0631

**Published:** 2025-11-06

**Authors:** Yuri Park, Jaeyeong Jeong, Eunsu Kim, Nuri Sung, Sang Jun Han

**Affiliations:** Department of Molecular and Cellular Biology, Baylor College of Medicine, Houston, USA

**Keywords:** SRC, endometriosis, PCOS, leiomyoma

## Abstract

Steroid receptor coactivators (SRCs) are master regulators of nuclear receptor signaling and play essential roles in female reproductive physiology. By integrating steroid hormone signaling with growth factors and metabolic pathways, SRC-1, SRC-2, and SRC-3 coordinate key processes such as decidualization, placental development, and ovarian function. Dysregulation of SRCs is strongly linked to the progression of benign gynecologic disorders, including polycystic ovary syndrome (PCOS), endometriosis, and uterine fibroids, largely through enhancing hormonal hypersensitivity and disrupting nuclear receptor–mediated cellular pathways. Emerging evidence further implicates specific SRC isoforms in disease pathogenesis, underscoring their potential as biomarkers and therapeutic targets. To inhibit SRC activity, natural compounds (e.g., gossypol, bufalin, verrucarin A) and synthetic small molecules (e.g., SI-2, SI-12, MCB-613) have been developed, demonstrating preclinical efficacy across several human diseases. However, their application in benign reproductive disorders remains largely unexplored. This review summarizes current knowledge of SRC biology in benign gynecologic disorders, outlines their mechanistic roles in disease progression, and highlights opportunities for clinical translation. Targeting SRCs may ultimately represent a novel, nonhormonal, fertility-preserving therapeutic strategy in women’s health.

## Introduction

Nuclear receptors (NRs) play a critical role in regulating female reproductive tissues in response to external hormonal stimuli. Accordingly, the expression and activity of NRs are tightly controlled in a tissue-specific manner. In this process, steroid receptor coactivators (SRCs) play a pivotal role by dynamically modulating interactions between NRs and transcription factors in response to extracellular hormonal signals (1, 2). By precisely regulating NR target gene expression, the SRC family maintains homeostasis and orchestrates diverse cellular responses to external environmental cues in female reproductive tissues.

**Figure 1:**
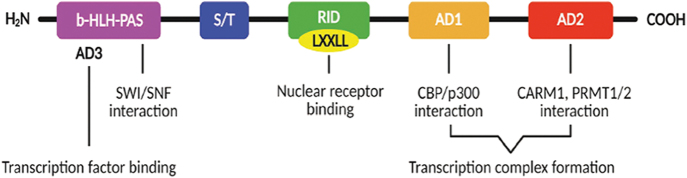
Structure and function of the domains of SRCs. All SRC family proteins contain three major functional regions. The N-terminal region includes the b-HLH-PAS domain and AD3. The b-HLH-PAS domain interacts with the SWI/SNF complex to mediate chromatin remodeling, while AD3, one of the SRC ADs, is essential for binding to other transcription factors. The RID domain contains the LXXLL motif, which directly binds NRs such as ER and AR. The C-terminal region consists of AD1 and AD2, which form transcriptional complexes through interactions with various coactivators and transcription factors. Abbreviations: b-HLH-PAS, basic helix-loop-helix/Per-ARNT-Sim; AD, activation domain; RID, nuclear RID.

SRCs consist of three proteins – SRC-1, SRC-2, and SRC-3 – each approximately 160 kDa in size. They share three core functional domains (Fig. 1): a basic helix-loop-helix/Per-ARNT-Sim (bHLH-PAS) domain, a nuclear receptor interaction domain (RID), and activation domains (ADs; AD1, AD2, and AD3). The bHLH-PAS domain is essential for protein–protein interactions and binding with other transcription factors. The RID contains the LXXLL motif, which directly interacts with NRs. The ADs recruit additional coactivators and transcription factors to assemble the transcriptional complex, thereby dynamically modulating NR function in response to external stimuli (1).

**Figure 2:**
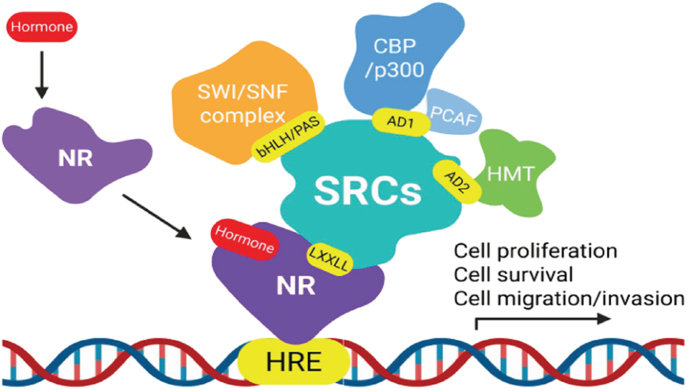
SRCs mediate transcriptional coactivation of NRs. Upon hormone exposure, NRs (ER, PR, and AR) bind to hormone response elements (HREs) within specific DNA sequences. The activated DNA-bound NR–hormone complex recruits SRC family proteins through interactions between the receptor’s LBD and the LXXLL motif of SRCs. SRCs then recruit additional coregulators, including histone acetyltransferases (e.g., CBP/p300 and PCAF), ATP-dependent chromatin remodeling complexes (e.g., SWI/SNF), histone methyltransferases (HMTs; e.g., CARM1 and PRMT1/2), and ubiquitin ligases, to dynamically and precisely regulate NR–target gene expression in response to hormonal signals.

NRs such as the estrogen receptor (ER), progesterone receptor (PR), and androgen receptor (AR) bind to specific DNA sequences known as hormone response elements (HREs) upon hormonal stimulation (3). The activated, DNA-bound nuclear receptor–hormone complex recruits SRCs through the interaction of the SRC LXXLL motif with the ligand-binding domain (LBD) of NRs (4, 5). Subsequently, SRCs recruit additional coregulators, including histone acetyltransferases (e.g., CBP/p300, PCAF), ATP-dependent chromatin remodeling complexes (e.g., SWI/SNF), histone methyltransferases (HMTs), and ubiquitin ligases, to dynamically and precisely regulate NR target gene expression in response to hormonal signals (Fig. 2) (4). In addition to NRs, SRCs interact with various intracellular signaling modulators, such as growth factor receptors (e.g., EGFR) and signaling kinases (e.g., MAPK, PI3K/Akt), to integrate and amplify cell proliferation signals through nongenomic pathways in response to external stimuli (6). For example, the splice variant of SRC-3, SRC-3Δ4, functions as a bridging adapter between EGFR and focal adhesion kinase (FAK), thereby promoting cancer cell migration and invasion in response to EGF stimulation (7).

Expression and activity of SRCs are tightly regulated to maintain normal cellular physiology in response to external stimuli, reflecting their essential role in NR-mediated processes. Consequently, dysregulation of SRCs is frequently associated with human disease progression (1). Based on previous studies identified through PubMed, we summarize here how SRCs regulate female reproductive tissue function, how their dysregulation contributes to the progression of female diseases, and how SRCs may be targeted therapeutically to treat these conditions.

## Differential expression profiles of SRCs in female reproductive tissues across the menstrual cycle

The menstrual cycle is a key regulator of female organ function, in part by controlling the expression and activity of essential molecular factors. In this context, SRC expression levels fluctuate across the menstrual cycle. In the human endometrium, SRC-1 protein expression is elevated in the glandular epithelium and stroma during the proliferative phase compared with the secretory phase, whereas SRC-2 and SRC-3 protein levels remain largely unchanged throughout the cycle (8, 9). However, another study reported no significant changes in SRC-1 or SRC-2 expression across the menstrual cycle in specific endometrial cell types, including the glandular epithelium, luminal epithelium, and stroma. In contrast, SRC-3 staining intensity was significantly increased in the glandular epithelium during the late proliferative phase compared with the early secretory phase (10). These discrepancies may arise from methodological differences, such as the use of distinct scoring systems for protein expression (e.g., H-score vs positivity index) or varying definitions of the secretory phase (e.g., total secretory phase vs early, mid, and late subdivisions). Ultimately, the differential hormonal environment across the estrous cycle modulates SRC expression levels, thereby influencing the cellular pathways under cycle-dependent regulation.

## Role of SRCs in female reproductive tissues

### SRCs in female fertility

As SRCs undergo cyclic expression changes in the endometrium, their roles in pregnancy and parturition become evident. For example, female SRC-1 knockout (KO) mice are fertile and exhibit normal growth compared with controls, although their uteri are smaller and lighter in response to hormonal stimulation (11). SRC-2 KO female mice exhibit reduced fertility compared with controls, characterized by fewer pups and smaller litter sizes during continuous mating (12). SRC-2 KO newborns are approximately 30% smaller and lighter than their WT littermates but catch up in growth by 3 months of age, indicating that SRC-2 is primarily required for normal development during the nursing period. Furthermore, PR-positive cell–specific SRC-2 KO female mice are infertile due to implantation defects and impaired decidualization in the uterus (13). The more severe infertility in PR positive cell-specific SRC-2 KO females likely reflects limited compensation compared with systemic KO mice. In systemic KOs, embryonic SRC-2 loss allows partial adaptation by other coactivators, such as SRC-1. In contrast, postpubertal SRC-2 deletion in PR-expressing tissues prevents such compensation, resulting in a distinct uterine-specific infertility phenotype.

Meanwhile, SRC-3 KO female mice are subfertile, as evidenced by significantly reduced pregnancy rates and smaller litter sizes compared with controls, and they also exhibit prolonged estrous cycles (14). SRC-3 KO female mice also produce significantly fewer eggs than controls, suggesting that SRC-3 is essential for maintaining ovarian function and supporting female fertility.

### Role of SRCs in decidualization of the uterus

Decidualization of the human endometrium involves the dramatic morphological and functional transformation of endometrial stromal cells into specialized decidual cells, a process essential for implantation of the fertilized egg. Because decidualization is critical for pregnancy, its impairment may contribute to subfertility or infertility observed in SRC KO mice. Given that estradiol (E2) and progesterone (P4) are key steroid hormones driving decidualization, the roles of SRCs in regulating their receptors (ER and PR) and associated transcriptional activity are likely indispensable. SRC-1 KO females are still fertile, but their decidual response is weaker than their control (15). In the endometrium, SRC-1 specifically binds to ERα during the proliferative phase, when ERα and E2 drive thickening of the uterine lining. This interaction indicates that SRC-1 contributes to mediating E2-dependent gene regulation in the endometrium (9). SRC-1 knockout reduces PR activity in the uterine stroma and myometrium following E2 + P4 treatment, accompanied by decreased expression of PR target genes such as *Il13ra2* and *Mig6* (16). These findings indicate that SRC-1 also promotes PR transcriptional activity in stromal and myometrial tissues. Therefore, SRC-1 KO mice exhibit decreased sensitivity to both estrogen and progesterone, along with a substantial reduction in decidualization (11).

SRC-2 KO mice exhibit altered expression of genes involved in PR, ER, Wnt, and BMP signaling pathways (17). Thus, PR-positive cell–specific SRC-2 KO female mice exhibit a reduced decidual response due to lower expression of key decidualization markers, including *Bmp2*, *Cox2*, and *Follistatin*, compared with controls. All these genes are direct PR targets (13, 18). In addition to findings in the mouse uterus, SRC-2 KO in human endometrial stromal cells also disrupts proper decidualization, indicating that SRC-2 plays an essential role in the P4-mediated decidualization process in the human endometrium (13, 18). Alongside enhanced progesterone signaling, metabolic reprogramming is a key driver of decidualization progression (19). During decidualization, glycolytic activity is markedly increased, generating sufficient lactate to sustain rapid cell proliferation and driving extensive metabolic remodeling required for the differentiation of stromal cells into decidual cells (18, 20). SRC-2 enhances glycolytic flux by upregulating fructose-2,6-bisphosphate (Fru-2,6-BP), a potent activator of glycolysis produced by 6-phosphofructo-2-kinase/fructose-2,6-bisphosphatase 3 (PFKFB3) (18). SRC-2 binds to the progesterone response element – containing region of the PFKFB3 promoter, thereby enhancing PFKFB3 expression, which is essential for proper decidualization. These findings suggest that SRC-2 is pivotal for endometrial decidualization by promoting metabolic reprogramming through glycolysis, a process likely essential for establishing a successful pregnancy.

SRC-3 plays a critical role in PR-mediated decidualization of the uterus, as SRC-3 KO female mice exhibit early pregnancy loss, reduced decidual size, and extensive decidual cell death (21). In addition, SRC-3 knockdown in primary human endometrial stromal cells (HESCs) significantly impairs the induction of key decidualization biomarkers and signatures (such as HAND2 and IL15) and prevents the morphological transformation of stromal cells into epithelioid decidual cells. RNA sequencing of SRC-3–deficient HESCs revealed downregulation of genes involved in chromatin remodeling, proliferation, migration, and programmed cell death, all of which play critical roles in decidualization.

### Role of SRCs in the placenta

SRCs play an important role in placental development and maintenance, processes that are essential for sustaining pregnancy and supporting fertility. SRC-1 exhibits cell-type-specific distribution within the placenta, being expressed in syncytiotrophoblasts, spongiotrophoblasts, endothelial cells, and various trophoblast populations within the labyrinth and junctional zones (22). Both SRC-1 and SRC-2 levels are reduced in placentas from early preterm births (22–29 weeks of gestation) compared with those from term deliveries (37–40 weeks) (23). Double knockout of SRC-1 and SRC-3 in female mice results in a marked reduction in embryo viability compared with single knockout females (22). Thus, SRC-1 may play a supportive role in placental development alongside SRC-3. However, its role in placental formation and function is likely nonexclusive.

SRC-2 knockout female mice exhibit reduced fertility, producing fewer pups and litters per female (12). Increased embryonic resorption in these mice contributes to the reduced number of pups. Embryos from SRC-2 knockout females frequently exhibit hypoplastic placentas characterized by fewer trophoblastic trabeculae and embryonic capillaries, indicating that SRC-2 is essential for normal placental development. A reduction in SRC-2 may therefore be associated with preterm birth due to its critical role in placental formation (12). Placental disorders such as preeclampsia often arise from inadequate migration and invasion of extravillous trophoblasts into the maternal uterus. SRC-2 has been shown to induce Wnt9A expression, which is essential for trophoblastic cell viability, motility, and invasiveness – functions that are critical for optimal placentation during early pregnancy in human extravillous trophoblast cell lines (24).

Compared with SRC-1 and SRC-2, SRC-3 mRNA expression remains unchanged; however, its protein level is reduced in early preterm placentas relative to term placentas (23). Therefore, dysregulation of post-translational modifications of SRC-3 is associated with the progression of preterm birth. Unlike SRC-2, however, SRC-3 does not affect the proliferation of human extravillous trophoblasts (24, 25). Instead, SRC-3 interacts directly with AKT in trophoblast cells and regulates migration and invasion through the AKT/mTOR signaling pathway (25). Under hypoxic conditions, accumulated HIF1α suppresses SRC-3 expression, thereby impairing the SRC-3/AKT/mTOR axis and increasing the risk of preeclampsia. In particular, reduced fetal vascular density and dilated maternal blood sinuses contribute to a markedly smaller labyrinth zone in SRC-1/SRC-3 double knockout placentas compared with single knockouts, demonstrating functional cooperation between SRC-1 and SRC-3 in regulating placental morphogenesis (22).

### Role of SRCs in the ovary

SRC-1 knockout female mice exhibit no overt ovarian structural or follicular abnormalities under basal conditions (26). SRC-2 is not required for ovulation, as both PR-positive cell–specific SRC-2 knockout and global SRC-2 knockout female mice produce a comparable number of oocytes following superovulation compared with controls (13). However, the absence of SRC-2 compromises uterine function; specifically, P4-mediated decidualization is severely impaired, underscoring its critical role in postovulatory implantation. In contrast, SRC-3 knockout female mice produce significantly fewer eggs than wild-type controls, although fertilization rates remain unaffected (14). This indicates that SRC-3 is essential for successful ovulation.

## Dysregulation of SRCs in the progression of benign female diseases

### Polycystic ovary syndrome (PCOS)

The levels and activities of SRCs are tightly regulated in female reproductive tissues due to their essential roles. Consequently, dysregulation of SRCs is frequently associated with the progression of benign female reproductive diseases.

Polycystic ovary syndrome (PCOS) is a common endocrine disorder affecting women of reproductive age. Patients with PCOS often experience infertility due to dysfunctional ovulation and hyperandrogenism. Altered expression of NRs has been reported in the endometrium of PCOS patients, potentially contributing to impaired fertility. Specifically, mRNA levels of ERα and AR are significantly elevated; however, only ERα protein expression is increased in the endometrium of PCOS patients compared with that of normal endometrium (27). In addition to NRs, SRCs are also dysregulated in PCOS. While SRC-1 expression remains comparable between normal and PCOS endometrium, SRC-2 and SRC-3 are markedly upregulated in the endometrium of PCOS patients (10, 27). ERα expression is significantly increased in both the epithelial and stromal compartments of the endometrium in PCOS patients (10). The concurrent upregulation of SRC-2 and SRC-3 alongside ERα suggests that estrogen signaling, particularly through the E2/ERα axis, is overactivated in the endometrium during PCOS. Elevated ERα levels, together with increased coactivators such as SRC-2 and SRC-3, may enhance steroid hormone sensitivity and transcriptional activity in the endometrium, thereby contributing to the progression of PCOS-associated endometrial dysfunction.

Beyond endometrial dysregulation, cumulus cells in PCOS exhibit distinct abnormalities that impair oocyte quality and disrupt normal follicle maturation (28). SRC dysregulation is also evident in cumulus cells. For example, in cumulus cells isolated from mature follicles of PCOS patients, SRC-1 expression is decreased, whereas SRC-2 and SRC-3 levels are increased compared with healthy controls (29). Therefore, the elevation of SRC-2 and SRC-3 may induce hormonal hypersensitivity in cumulus cells, disrupting communication between cumulus cells and oocytes and impairing ovarian function. A hallmark of PCOS is abnormal folliculogenesis, characterized by an increased number of small, immature follicles and elevated levels of androgens and luteinizing hormone (30). In granulosa cells of these immature follicles, however, SRC-1 protein levels are markedly elevated in PCOS patients compared with controls (31). Therefore, SRC-1 is misregulated in a cell type-specific manner in PCOS follicles. In addition, SRC-2 and SRC-3 levels are elevated in granulosa cells of immature follicles from PCOS patients compared with controls (Table 1) (32).

**Table 1:** Steroid receptor coactivators in women’s benign reproductive diseases.

SRC member	Disease	Molecular mechanism	Clinical implication	References
SRC-1	PCOS	SRC-1 expression is reduced in cumulus cells from PCOS patients but significantly elevated in granulosa cells of immature follicles	Reduced SRC-1 expression in cumulus cells may impair oocyte maturation and follicular competence, whereas increased SRC-1 enhances ER transactivation in granulosa cells, leading to follicular arrest. May contribute to infertility and chronic anovulation	(29, 31)
	Endometriosis	Colocalizes with ERα to boost estrogen signaling and CXCL12; 70 kDa isoform blocks TNF-α apoptosis via procaspase-8/ERβ and promotes EMT	Reduced in the secretory-phase endometrium of endometriosis patients; 70 kDa isoform promotes lesion survival, invasion, and progression by blocking cell death	(8, 34, 35, 37, 73)
	Uterine fibroids	Upregulated in fibroids; coactivates ERα/PR; recruited to KLF11 promoter by mifepristone, activating TGF-β signaling	Recruited to PR targets by selective progesterone receptor modulator (SPRMs), suppressing fibroid growth	(42, 43, 49)
SRC-2	PCOS	Overexpressed in endometrial cells, enhancing ERα/AR signaling and estrogen sensitivity	Drives PCOS-related endometrial hyperplasia, infertility, and cancer risk	(10, 27, 29, 30, 32)
		Overexpressed in the cumulus and granulosa cells	Induces hormonal hypersensitivity in the ovary; disrupts cumulus–oocyte communication; contributes to abnormal follicle maturation	
	Endometriosis	Required for P4 suppression of E2-induced CXCL12 in endometriosis; expression is unchanged between cases and controls	Knockout reduces lesion size in mice	(8, 34, 35)
	Uterine fibroids	Overexpressed in ∼50% of fibroids; coactivates ER/PR; recruited with SRC-1 to KLF11 by mifepristone	Essential for progesterone signaling; activation with SPRMs can suppress fibroid growth	(41, 49)
SRC-3	PCOS	SRC-3 is upregulated in epithelial and stromal cells, disrupting cyclic estrogen withdrawal in secretory-phase endometrium	Overactive estrogen signaling drives endometrial dysfunction in PCOS	(10, 27, 29, 30, 32)
		Overexpressed in the cumulus and granulosa cells	Induces hormonal hypersensitivity; disrupts cumulus–oocyte communication; contributes to abnormal follicle maturation	

### Endometriosis

Endometriosis is an estrogen-dependent inflammatory disease characterized by the ectopic growth of endometrial-like tissue outside the uterus, including the ovaries, intestines, and peritoneal cavity (33). Endometriosis affects up to 10% of women of reproductive age and is associated with chronic pelvic pain and infertility. SRC-1 plays an essential role in disease progression, as endometriotic lesions fail to develop in SRC-1 knockout female mice following surgically induced endometriosis (34). Therefore, both the expression levels of SRC-1 and the number of SRC-1–positive cells in ovarian endometriosis are higher than those of SRC-2 and SRC-3 (8). C-X-C motif chemokine ligand 12 (CXCL12) expression is elevated in ovarian endometriosis across all menstrual phases, playing a key role in promoting the proliferation, migration, and invasion of endometriotic cells (35, 36). SRC-1 knockout significantly reduces E2-induced CXCL12 expression in endometriotic stromal cells, suggesting that SRC-1 is a key enhancer of cytokine signaling in these cells. In addition, ERα colocalizes with SRC-1 in most glandular and many stromal cells of ovarian endometriotic tissue (8). Given the essential role of ERα in the progression of endometriosis, the SRC-1/ERα axis may act as a key mechanistic driver of disease development. Interestingly, SRC-1 undergoes a conformational change that facilitates the progression of endometriosis. Compared with normal endometrium, the 70 kDa SRC-1 isoform is highly elevated in endometriotic lesions relative to full-length SRC-1. This isoform is generated from full-length SRC-1 by proteolytic cleavage mediated by activated matrix metalloproteinase-9 (MMP-9), which is also elevated in endometriotic lesions. The SRC-1 isoform interacts with procaspase-8 and ERβ, inhibiting TNF-α–induced apoptosis by disrupting the caspase-8/caspase-3/Bid pathway in immortalized endometrial epithelial cells (34, 37). Furthermore, it promotes cellular invasion by upregulating epithelial–mesenchymal transition (EMT) markers such as N-cadherin, vimentin, Slug, Snail, β-catenin, and TCF8 (34).

SRC-2 and SRC-3 also play essential roles in endometriosis progression, as knockout of either results in significantly smaller lesions in mouse models compared with controls (34). However, mechanistic studies defining the roles of SRC-2 and SRC-3 in endometriosis progression have not yet been conducted. SRC-2 plays a critical role in metabolic regulation, whereas SRC-3 is actively involved in inflammation (38, 39). Therefore, the roles of SRC-2 and SRC-3 in the dysregulation of metabolism and immune responses associated with endometriosis warrant further investigation (Table 1).

### Uterine fibroids (leiomyomas)

Uterine fibroids are the most common benign tumors that develop within or on the uterus. Although their exact etiology remains unclear, estrogen is widely recognized as a key driver of fibroid growth (40). Fibroids exhibit heightened sensitivity to estrogen, partly due to the induction of estrogen-responsive target gene transcription. This estrogen hypersensitivity suggests that SRCs may contribute to the pathogenesis of fibroids (41). Thus, SRC-1 upregulation has also been observed in fibroids compared with the myometrium (42, 43). In addition, other studies have reported SRC-2 and SRC-3 overexpression in 50 and 40% of uterine fibroid cases, respectively, compared with adjacent myometrium, suggesting a potential pathological role for these coactivators in fibroid development (41). At the NR level, microvascular endothelial cells constitutively express ERβ, whereas myometrial smooth muscle cells (SMCs) primarily express ERα in both fibroid and myometrial tissues (44, 45). This distribution pattern suggests that ERα may drive fibroid formation, whereas ERβ may promote vascularization to support the growth of fibroids. In addition, SRC-1, SRC-2, and SRC-3 are coexpressed with ERα in leiomyoma SMCs (46). Therefore, functional activation of SRCs/ERα may serve as a mechanistic driver of uterine fibrosis. However, despite these observations, no significant correlation has been identified between SRC expression levels and ER subtypes in fibroids (45). Elevated levels of PR-A and PR-B have been detected in leiomyomas compared with normal myometrium, where they promote tumor proliferation by upregulating growth factors (EGF and IGF-1) and activating the PI3K/AKT and MAPK pathways (47, 48). Interestingly, the selective PR modulator RU486 (mifepristone) suppresses uterine fibroid progression by promoting the recruitment of SRC-1 and SRC-2 to both the basal promoter and a distal PR–binding region of the tumor suppressor gene KLF11 (49). SRCs also play a critical role in drug-mediated suppression of uterine fibroid progression. Collectively, these findings suggest that SRC family members may contribute to fibroid pathogenesis through both estrogen- and progesterone-dependent mechanisms (Table 1).

## Therapeutic implications of targeting SRC inhibitors for the treatment of women’s benign diseases

### Natural products targeting SRCs

Gossypol, a natural polyphenolic compound derived from the cotton plant, has emerged as a potent inhibitor of the SRCs SRC-1 and SRC-3. In ERα-positive MCF-7 breast cancer cells, gossypol selectively suppresses the expression of SRC-1 and SRC-3 proteins in an ERα ligand-independent manner (50). Mechanistically, gossypol binds directly to the RID of SRC-3, leading to its degradation in multiple cancer cell lines. This inhibition reduces cell viability and enhances sensitivity to growth factor pathway inhibitors. Clinically, gossypol reduces the size of endometriotic lesions and uterine fibroids, with amenorrhea occurring in approximately 80% of treated patients (51). *In vitro* studies show that gossypol inhibits the proliferation of deep ovarian endometriosis cells at concentrations of 25–50 nM, although its potential mitochondrial toxicity remains a concern (52). However, gossypol is not currently approved by the U.S. FDA for any medical use because it has hepatotoxicity and cardiotoxicity in both animal models and humans (53, 54). To overcome this, low-toxicity derivatives of gossypol are currently under development (55).

Bufalin exhibits high potency with a low IC_50_, binding directly to the RID of SRC-3 and inducing proteasome-mediated degradation (56). Bufalin enhances the sensitivity of MCF-7 cells to the AKT inhibitor MK-2206 and suppresses cell proliferation. In ovarian cancer, bufalin inhibits the mTOR/HIF-1α pathway, while in colon cancer it reduces the expression of MIF, c-Myc, and HIF-1α and suppresses M2 macrophage polarization (57, 58, 59). In endometriosis, bufalin disrupts the SRC-1/ERβ axis by inhibiting the transcriptional activity of the SRC-1 isoform and promoting ERβ degradation through PSMD2- and UBA7-mediated proteasomal pathways (60). In mouse models, bufalin treatment reduces endometriotic lesion volume by inducing apoptosis and pyroptosis while preserving normal uterine function, indicating minimal cytotoxicity. However, bufalin is not currently approved by the U.S. FDA for any medical use due to its cardiotoxicity (61). To overcome this issue, new derivatives of bufalin, such as acetyl-bufalin, have been developed with reduced toxicity for clinical application (62).

Verrucarin A, a fungal metabolite, also demonstrates potent inhibition of SRCs. It suppresses the intrinsic transcriptional activities of all three SRCs and impairs SRC-mediated ER transcription (63). At low concentrations, verrucarin A selectively reduces SRC-3 protein levels and inhibits the growth of various cancer cells, including MCF-7 (63). Moreover, low-dose verrucarin A sensitizes MDA-MB-231 and T-47D breast cancer cells to paclitaxel and tamoxifen, suggesting potential for combination therapy (63). These findings highlight the therapeutic potential of targeting SRC-1 and SRC-3 in gynecological diseases, despite the lack of direct evidence regarding the effects of verrucarin A on women’s reproductive disorders. However, verrucarin A exhibits severe multi-organ toxicity (64). To reduce toxicity, strategies such as conjugating verrucarin A to antibodies (antibody–drug conjugates) or employing folate-targeted delivery systems have been investigated for clinical application (65, 66).

### Synthetic small molecules targeting SRCs

The small-molecule SRC-3 inhibitor SI-2 is a selective inhibitor of SRC-3 that also reduces the protein levels and transcriptional activities of all three SRC family members. SI-2 binds directly to the RID of SRC-3 and selectively suppresses SRC-3 protein expression at low concentrations (67). In both *in vitro* and *in vivo* models, SI-2 selectively inhibits the proliferation and migration of breast cancer cells by targeting SRC-3 activity while exerting minimal effects on non-cancerous cells and showing no detectable acute or chronic toxicity. Beyond its tumor-intrinsic effects, SI-2 also modulates the tumor-associated immune microenvironment by regulating SRC function in immune cells. For example, SI-2 inhibits SRC-3 function in regulatory T cells (Tregs), leading to a loss of their immunosuppressive activity (39). In immune-competent mouse models, SRC-3 inhibition by SI-2 treatment significantly reduces mammary tumor growth and lung metastasis, accompanied by increased infiltration of cytotoxic CD4^+^ and CD8^+^ T cells and CD56^+^ natural killer cells into tumors (68) (Table 2).

**Table 2:** Small molecules targeting steroid receptor coactivators.

Inhibitor	Target(s)	Mechanism	Application	Key findings	References
Gossypol	SRC-1, SRC-3	Binds SRC-1/3 RID, reducing cell viability and disrupting ER/PR activity	Breast, prostate, endometrial cancers; endometrioma; fibroids	First proof-of-concept SRC inhibitor; micromolar IC_50_; synergizes with growth factor inhibitors	(50, 51, 52, 74)
Bufalin	SRC-3, SRC-1	Binds SRC-1/3 RID and triggers proteasomal degradation	Breast, ovarian, colorectal cancers; endometriosis	High potency (low-nM IC_50_); synergizes with chemotherapy	(56, 58, 59, 60, 75, 76)
Verrucarin A	SRC-1, SRC-2, SRC-3	Inhibits SRC via transcriptional interference, lowering SRC-3 at low doses	Preclinical cancer models	Identified by high-throughput screening; disrupts SRC-driven transcription	(63)
SI-2	SRC-1, SRC-2, SRC-3	Directly binds SRC-3 RID, lowering protein levels and transcriptional activity	Breast cancers	IC_50_ 3–20 nM; inhibits tumors *in vivo* with low toxicity; synergizes with endocrine/targeted therapy; modulates immune microenvironment	(39, 67, 68, 77)
SI-10	SRC-3	SI-2 derivatives with greater potency, specificity, and stability bind SRC-3 and lower its protein level	Breast cancer, prostate cancer, mantle cell lymphoma	Second-generation inhibitor; synergizes with other inhibitors	(69, 70, 71)
SI-12					
MCB-613	SRC-1, SRC-2, SRC-3	Enhances all SRCs; binds SRC-3 RID, hyperactivates, then drives proteasomal degradation	Breast cancer	SRC coactivators: induces oxidative and ER stress, which triggers cell death	(78)

The second-generation inhibitor SI-12 was developed by modifying the SI-2 scaffold with fluorine substitutions, enhancing potency, SRC-3 specificity, and pharmacokinetics (68). Compared to SI-2 (with a ∼1 h plasma half-life), SI-12 significantly improves bioavailability, achieving a plasma half-life of over 20 h (69). SI-12 primarily binds to SRC-3 and downregulates its protein levels, thereby suppressing breast cancer cell proliferation, migration, and invasion *in vitro* and *in vivo*, with minimal cytotoxicity to normal cells. In breast cancer PDX-derived organoids, SI-12 significantly inhibits tumor growth (70). Although their antitumor effects have not been extensively investigated across all cancer types, these compounds have demonstrated efficacy in breast cancer, prostate cancer, and mantle cell lymphoma (69, 70, 71) (Table 2).

Unlike SI-family inhibitors, MCB-613 enhances the transcriptional activity of all three SRCs without increasing their protein expression (72). MCB-613 binds to the RID of SRC-3 and recruits additional coactivators, leading to increased oxidative and endoplasmic reticulum stress that activates Abl kinase signaling. This hyperactivation ultimately drives proteasomal degradation of SRC-3 (72). As a result, MCB-613 induces cell death predominantly through paraptosis and effectively suppresses mammary tumor growth *in vivo*. These findings suggest that paradoxical hyperactivation of SRC-3 may represent a viable therapeutic strategy by overwhelming cancer cell stress responses and triggering cell death (Table 2).

Unfortunately, no reports are available on the use of these synthetic small molecules as therapies for benign female reproductive diseases, despite the critical role of SRCs in the development of these conditions. In addition, no reports of cytotoxicity for SI-2, SI-12, or MCB-613 have been published to date.

## Future directions and clinical translation

Translating SRC biology into clinical practice for benign female reproductive diseases remains incomplete, with significant gaps in knowledge. Deeper mechanistic studies of SRC-1, SRC-2, and SRC-3 using tissue-specific models and patient-derived samples are needed. In addition, investigations into disease-specific isoforms of SRCs may reveal novel therapeutic opportunities.

Although SRC inhibitors and degraders such as gossypol, bufalin, and SI-2 have shown promise in cancer, their potential in benign reproductive diseases remains unexplored. To address this, studies employing disease-specific organoid systems and animal models are essential to evaluate the efficacy and safety of these SRC-targeting compounds.

Ultimately, SRC-directed therapies are likely to be most effective when combined with hormonal or metabolic modulators. Defining such combinations could pave the way toward non-hormonal, fertility-preserving treatments that overcome the limitations of current therapies. Bridging mechanistic discoveries with translational studies will be key to establishing SRCs as novel therapeutic targets in women’s health.

## Conclusion

SRC-1, SRC-2, and SRC-3 are central regulators of female reproductive physiology, and their dysregulation contributes to the progression of benign gynecologic disorders such as PCOS, endometriosis, and uterine fibroids. Isoform- and context-specific actions of SRCs underscore their complexity in disease progression, while also highlighting their potential as biomarkers and therapeutic targets. In this context, small-molecule SRC inhibitors present promising opportunities for developing non-hormonal therapies in women’s reproductive health.

## Declaration of interest

The authors declare that there is no conflict of interest that could be perceived as prejudicing the impartiality of this work.

## Funding

This work was supported by the *Eunice Kennedy Shriver* National Institute of Child Health and Human Development (R01-HD098059) to Sang Jun Han and by the Department of Defense’s Congressionally Directed Medical Research Programs (HT9425-24-1-0254) awarded to Sang Jun Han.

## Author contribution statement

Yuri Park and Jaeyeong Jeong conceptualized the study and drafted the manuscript. Eunsu Kim and Nuri Sung reviewed and discussed the manuscript. Sang Jun Han organized and revised the manuscript.
